# Intranasal Delivery of Cholera Toxin Induces Th17-Dominated T-Cell Response to Bystander Antigens

**DOI:** 10.1371/journal.pone.0005190

**Published:** 2009-04-10

**Authors:** Jee-Boong Lee, Ji-Eun Jang, Man Ki Song, Jun Chang

**Affiliations:** 1 Division of Life and Pharmaceutical Sciences, and Center for Cell Signaling & Drug Discovery Research, Ewha Womans University, Seoul, Republic of Korea; 2 Laboratory Science Division, International Vaccine Institute, Seoul, Republic of Korea; New York University School of Medicine, United States of America

## Abstract

Cholera toxin (CT) is a potent vaccine adjuvant, which promotes mucosal immunity to protein antigen given by nasal route. It has been suggested that CT promotes T helper type 2 (Th2) response and suppresses Th1 response. We here report the induction of Th17-dominated responses in mice by intranasal delivery of CT. This dramatic Th17-driving effect of CT, which was dependent on the B subunit, was observed even in Th1 or Th2-favored conditions of respiratory virus infection. These dominating Th17 responses resulted in the significant neutrophil accumulation in the lungs of mice given CT. Both *in vitro* and *in vivo* treatment of CT induced strongly augmented IL-6 production, and Th17-driving ability of CT was completely abolished in IL-6 knockout mice, indicating a role of this cytokine in the Th17-dominated T-cell responses by CT. These data demonstrate a novel Th17-driving activity of CT, and help understand the mechanisms of CT adjuvanticity to demarcate T helper responses.

## Introduction

Cholera toxin (CT), a major enterotoxin produced by *Vibrio cholerae*, is a potent mucosal immunogen as well as adjuvant that enhances mucosal and systemic antibody responses to codelivered antigens [1]. Most studies have proposed that CT promotes a strong Th2-dominant response to bystander antigens, based on the production of IL-4, IL-5, and IL-10 but little IFN-γ [2]–[5]. Furthermore, it has been also shown that CT and heat-labile enterotoxin from *Escherichia coli* (LT) can suppress IL-12 and IFN-γ production [6], [7]. However, other studies have reported mixed Th1/Th2 response with the production of both IFN-γ and IL-4 following oral and intranasal immunization with CT [8], [9], and Lavelle *et al.* demonstrated that CT also promotes the generation of regulatory T cells against bystander antigens [10]. Thus, it remains to be determined whether adjuvanticity of CT may extend to the induction of other T-cell subsets such as recently described IL-17-producing Th17 cells.

In this study, we examined the possibility that CT exerts any regulatory effect on the differentiation of IL-17-producing CD4 T cells in murine models. Here we demonstrate a novel ability of CT that induces strong Th17-type responses against CT as well as bystander antigens through intranasal delivery.

## Results

### Co-delivery of CT induces IL-17-producing CD4 T cells to bystander antigen

**Figure 1:**
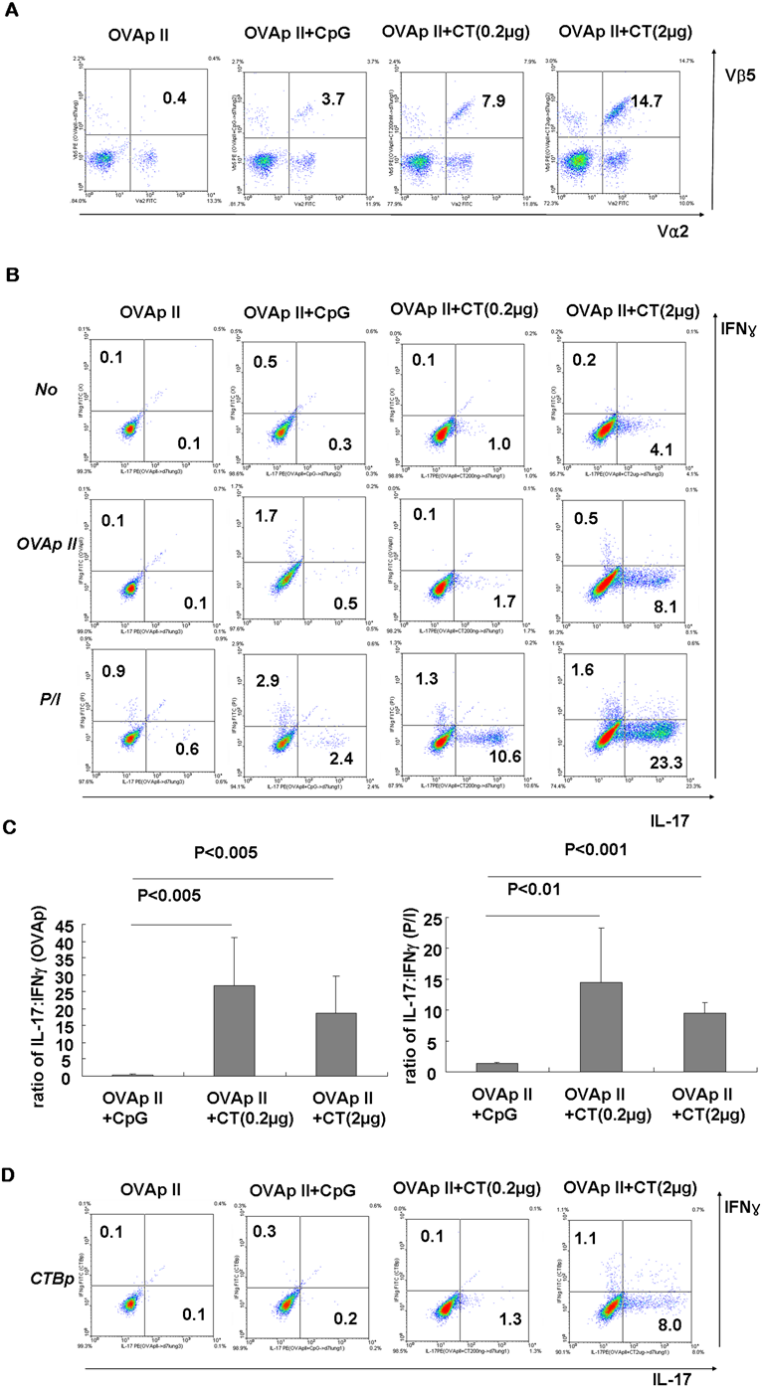
Co-delivery of CT induces IL-17-producing CD4 T cells to bystander peptide antigen. After transfer of OT-II splenocytes, the recipient B6 mice were immunized once with 40 µg of OVAp II together with CpG (30 µg) or CT (0.2 µg or 2 µg) intranasally. (A) Lung tissues were isolated at day 7 and the frequencies of donor OT-II cells were measured by CD4, Vα2 and Vβ5 staining. (B) The lung mononuclear cells were stimulated with OVAp II or PMA/ionomycin, or CTB peptide (D) and stained for CD4, IFN-γ and IL-17. Only CD4^+^ gated cells were shown in the dot plots. (C) The graphs indicate the average ratio of IL-17:IFN-γ expression of CD4^+^ gated cells. Dot plots are representative of at least three independent experiments and data are average ± SEM, n = 4∼5 mice per group.

To investigate whether CT has any regulatory activity on the differentiation of Th17 cells, B6 mice were transferred with naïve TCR-transgenic OT-II cells and then immunized once with OVA_323-339_ peptide (ISQAVHAAHAEINEAGR; herein referred to as OVAp II) together with CT intranasally. For comparison, CpG oligodeoxynucleotide (ODN) was employed, which is well known to promote T helper type 1 response [11]. At the peak responses (day 7), ∼7.9% and ∼14.7% were Vα2^+^Vβ5^+^ donor OT-II cells among CD4-gated cells in the lungs of OVAp II+CT(0.2 µg) and OVAp II+CT(2 µg) group, respectively, while ∼3.7% in the OVAp II+CpG group (Fig. 1A). The cytokine profiles of CD4^+^ T cells from the lungs of OVAp II+CT group revealed significantly increased expression of IL-17 in response to stimulation with OVAp II or PMA/ionomycin (P/I) with dose-dependent manner, when compared to OVAp II or OVAp II+CpG group (Fig. 1B). As a negative control, OVAp II alone had no effect on the differentiation of Th cells. The average ratio of IL-17:IFN-γ-producing cells clearly indicates that OVA-specific CD4 T cells were undergoing skewed differentiation exclusively toward Th17 with co-delivery of CT (Fig. 1C). CT is also one of the most powerful mucosal immunogens and the B subunit of CT (CTB) contains an immunodominant CD4 T-cell epitope, CTB_89-100_ peptide (NNKTPHAIAAIS; herein referred to as CTBp), well-recognized by T cells of H-2*^b^* background [12]. So, we checked CTBp-specific CD4 T-cell response in the lungs of immune mice by intracellular cytokine staining, and found that ∼1.3% and ∼8.0% of gated CD4 T cells produce IL-17 upon CTB peptide stimulation for OVAp II+CT(0.2 µg) and OVAp II+CT(2 µg) group, respectively (Fig. 1D). The ratio of IL-17:IFN-γ-producing cells by CTB peptide stimulation was similar to that by PMA/ionomycin stimulation. However, in this single immunization setting, neither any significant IL-4 or IL-10 production was detected *ex vivo* by ELISA or intracellular cytokine staining in the lungs of either group of mice, nor any significantly elevated Th17 response in the spleen and mediastinal lymph nodes of OVAp II+CT group (data not shown). These results clearly demonstrate that single intranasal co-delivery of CT induces Th17-dominated T-cell responses to CT as well as bystander antigen in the lungs.

**Figure 2:**
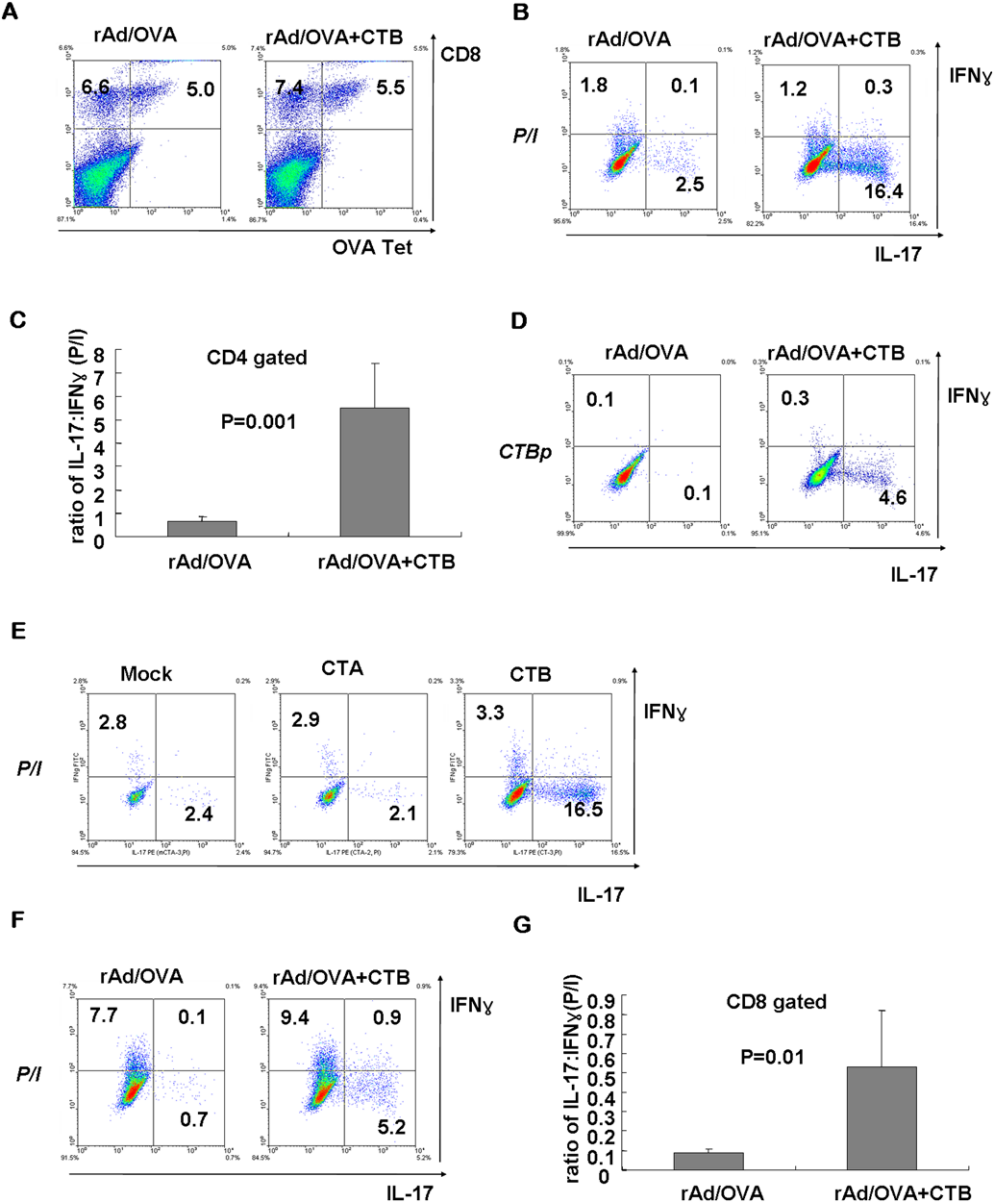
Intranasal CT co-delivery induces IL-17-producing CD4 T cells to bystander antigen expressed by recombinant adenovirus. B6 mice were injected with the rAd/OVA together with PBS or 2 µg of CTB intranasally. At day 14 after immunization, lung tissues were isolated and the frequencies of OVA-specific CD8 T cells were measured by anti-CD8 and OVA-Tet staining (A). The lung mononuclear cells were stimulated with PMA/ionomycin (B), or CTB peptide (D) and stained for CD4, IFN-γ and IL-17. CD4^+^ gated cells were shown in the dot plots. (C) The values of graph represent the average ratio of IL-17:IFN-γ-producing CD4 cells. (E) B6 mice were injected with recombinant CTA (10 µg) or CTB (2 µg) intranasally and at day 7 the lung mononuclear cells were stimulated with PMA/ionomycin. Only CD4^+^ gated cells were shown. (F) The same lung cells stimulated with PMA/ionomycin as in Fig. 2B were also stained for CD8, IFN-γ and IL-17. CD8^+^ gated cells were shown in the dot plots. (G) The graph represents the average ratio of IL-17:IFN-γ-producing CD8^+^ cells. Dot plots are representative of at least two independent experiments and data are average ± SEM.

To confirm whether the skewed differentiation of Th17 subset by mucosal CT delivery also occurs in other settings of immunization, we employed respiratory inoculation of recombinant defective adenovirus expressing OVA protein (rAd/OVA). We also made use of CTB instead of whole CT to see whether Th17-inducing effect of CT resides in the B subunit. At day 14 after rAd immunization, the frequencies of CD8^+^Tet^+^ cells infiltrating the lungs of rAd/OVA+CTB recipients were similar to those of rAd/OVA group (∼5.5% vs. ∼5.0%), although the absolute numbers of CD8^+^Tet^+^ cells were higher in rAd/OVA+CTB group (∼3×10^5^ vs. ∼1.8×10^5^; Fig. 2A). When the CD4 T cells were stimulated with PMA/ionomycin and stained for IL-17 and IFN-γ, rAd/OVA+CTB group showed a significantly increased ratio of IL-17:IFN-γ-producing cells, compared to rAd/OVA group (Fig. 2B and 2C). Consistent with the previous results with peptide immunization shown in Fig. 1, a similar ratio of IL-17:IFN-γ-producing cells was also observed upon stimulation with CTB peptide (Fig. 2D). To exclude the possibility that Th17-driving activity of CTB is mediated by contaminated CT A subunit in the CTB preparation, 10 µg of purified recombinant CTA was used in similar experiments and the results have shown that CTA has little Th17-driving activity (Fig. 2E). Taken together, these results strongly suggest that CT B subunit attributes to the Th17-skewing activity of CT.

Recently, the existence of IL-17-secreting CD8 T-cell subset (Tc17) was reported, which could be induced by TGF-β and IL-6 [13] or T-bet/Eomesodermin double knockout condition [14]. Thus, we checked whether IL-17-producing CD8 T-cell responses were also induced by mucosal CTB delivery with rAd inoculation. Although the frequency of IFN-γ-positive cells was still higher than that of IL-17-positive cells among CD8^+^ T cells, IL-17 producers were clearly increased in the lungs of rAd/OVA+CTB recipients compared to rAd/OVA recipients (Fig. 2F), and the average ratio of IL-17:IFN-γ-producing CD8 T cells was also significantly increased (Fig. 2G). These results indicate that CTB also influences the differentiation of CD8 T cells and induces Tc17-type CD8 T-cell responses.

### Th17-driving ability of CT upon Th1 or Th2-favored pulmonary RSV infection

**Figure 3:**
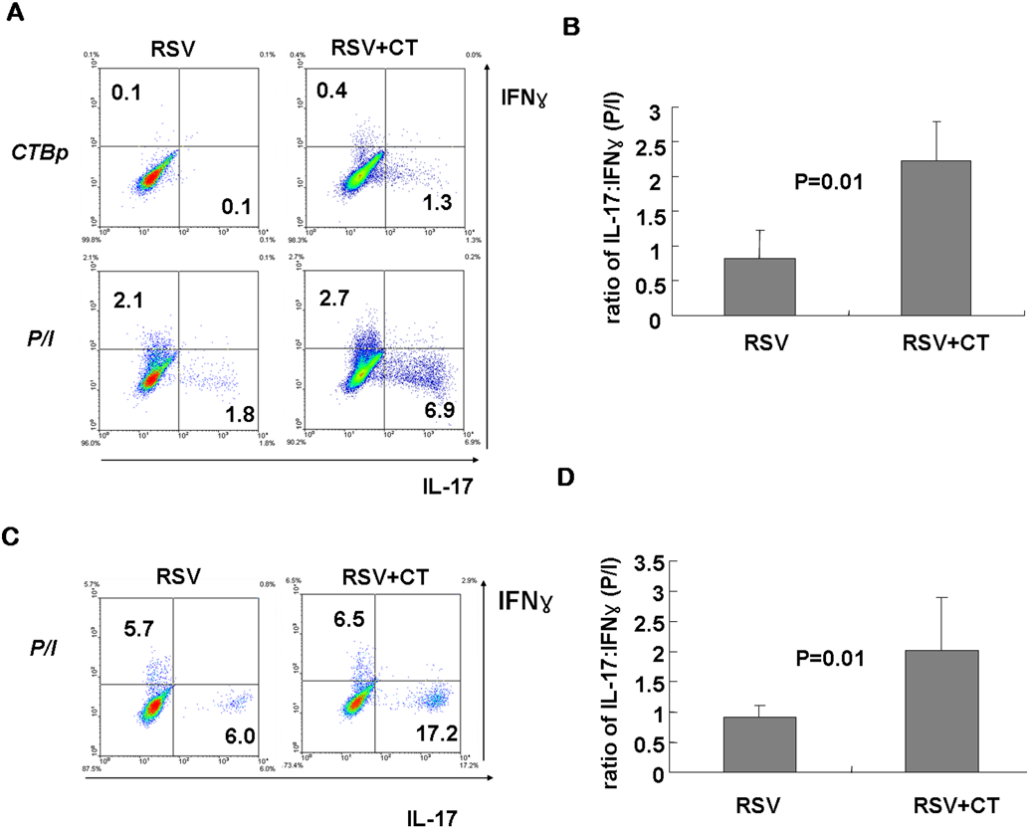
Th17-driving ability of CT upon pulmonary RSV infection in B6 and BALB/c mice. (A) B6 mice were intranasally infected with live RSV together with PBS or CT. At day 7 after infection, lung tissues were isolated, stimulated with PMA/ionomycin, and stained for CD4, IFN-γ, and IL-17. CD4^+^ gated cells were shown in the dot plots. (B) The graph represents the average ratio of IL-17:IFN-γ-producing CD4 cells. (C, D) The similar experiments were performed in BALB/c mice. Dot plots are representative of at least two independent experiments and data are average ± SEM, n = 4∼5 mice per group.

B6 mice are generally predisposed to produce Th1-biased responses to respiratory syncytial virus (RSV) infection [15] and other pathogens [16], [17]. To examine whether CT delivery still has Th17-driving ability in the Th1-favored environment, we employed RSV infection model in B6 mice and compared T-cell responses in the presence or absence of CT. At the peak responses, the percentages of IL-17-producing CD4 T cells in the lungs of RSV+CT group were higher than RSV group (∼6.9% vs. ∼1.8%; Fig. 3A), and the average ratio of IL-17:IFN-γ-producing CD4 T cells was also significantly increased (Fig. 3B). Consistent with the previous results, ∼1.3% was CTBp-specific among gated CD4 T cells as judged by peptide stimulation (Fig. 3A).

We also examined the Th17-driving ability of CT upon RSV infection in BALB/c mice, which are prone to develop Th2 rather than Th1. Similar to B6 mice, Th17-skewed responses were observed upon pulmonary RSV infection in the presence of CT (Fig. 3C and D). The total percentages of IL-17 and/or IFN-γ-producing CD4 T cells are generally higher in BALB/c mice than B6 mice upon RSV infection. Together, these results clearly demonstrate that even Th1 or Th2-prone response to respiratory viral infection could be redirected to Th17-skewed one by CT and Th17-driving ability of CT is independent of H-2 background.

### CT induces neutrophil recruitment in the lung

**Figure 4:**
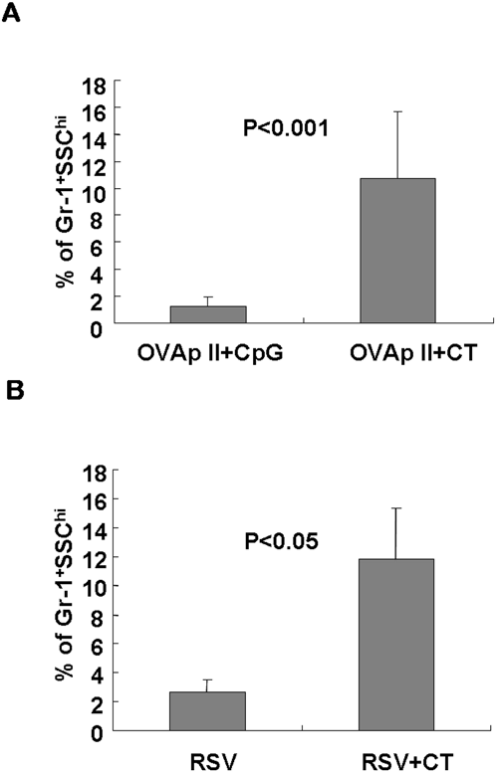
CT induces neutrophil recruitment in the lungs. (A) B6 mice were given 40 µg of OVAp II peptide intranasally with 30 µg of CpG or 2 µg of CT. Lung tissues were isolated at day 7, and the number of neutrophils was measured by Gr-1 staining and side scatter (SSC) values. (B) BALB/c mice were inoculated with RSV in the presence or absence of CT and the percentages of neutrophils are determined as in (A). Data are obtained from two independent experiments and are average ± SEM, n = 4∼5 mice per group.

Proinflammatory IL-17 is thought to promote inflammatory responses by regulating production of chemokines and recruiting neutrophils [18]–[20]. To test whether Th17 cells induced by CT trigger airway infiltration of neutrophils, we examined neutrophil recruitment in the lung tissues of B6 mice with anti-Gr-1 staining at day 7 after peptide immunization. The neutrophils represent the vast majority of Gr-1(Ly-6G)-positive cells in the periphery, although Gr-1 is also expressed on some granulocytes including eosinophils [21]. OVAp II+CT mice exhibited excessive Gr-1^+^ neutrophil infiltration in the lung airway, compared with the OVAp II+CpG group (Fig. 4A). We also examined the lungs of BALB/c mice infected with RSV+CT, and consistently observed significantly increased numbers of Gr-1^+^ neutrophils compared to the RSV group (Fig. 4B). These data suggest that Th17 response induced by CT delivery promote recruitment of neutrophils possibly by regulating the production of cytokines/chemokines and/or other cells involved in neutrophil accumulation.

### CT-induced IL-6 production, which is dependent on GM1 binding, is required for Th17-driving activity

**Figure 5:**
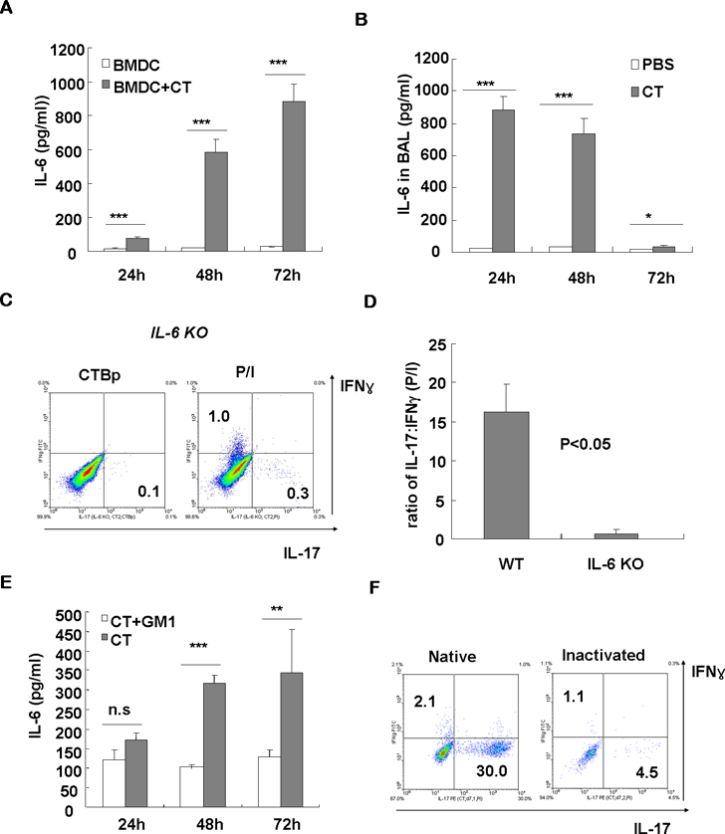
CT induces IL-6 production *in vitro* and *in vivo*, which is required for Th17-driving activity. (A) Dendritic cells were generated from bone marrow of BALB/c mice by culturing with GM-CSF and IL-4. After 6 days, DCs were purified and cultured in the presence or absence of CT. At indicated time points, the levels of IL-6 cytokine in the supernatant were determined by ELISA. (B) B6 mice were intranasally injected with 2 µg of CT and BAL samples were prepared at indicated time points. The levels of IL-6 in the BAL fluids were measured by ELISA. (C, D) IL-6KO mice with B6 background were intranasally injected with CT, and lung mononuclear cells were prepared at day 7 and IL-17- and IFN-γ-producing CD4 T cells were measured by ICS upon stimulation. (E) For blocking experiment, CT was pre-incubated with 5-fold molar excess of ganglioside GM1 and then added to the DC culture. Data are average ± SEM, and representative of two experiments. (F) CT was heat-inactivated by boiling for 30 min and intranasally administered into normal B6 mice. At day 7, lung tissues were isolated, stimulated with PMA/ionomycin, and stained for CD4, IFN-γ, and IL-17. *, P<0.05; **, P<0.01; ***, P<0.001; n.s., not significant.

The cytokines thought to be critical for promoting murine Th17 differentiation are IL-6 and TGF-β [22]–[24]. The findings that CT actively induces Th17 differentiation and airway neutrophilia suggest that IL-6 and/or TGF-β could be involved in the skewed Th17 differentiation by CT. We thus examined whether the expression of IL-6 and/or TGF-β could be induced by CT using bone marrow-derived dendritic cells *in vitro*, since CT has been found to directly affect cytokine profiles of APC [7], [25]. Significantly increased IL-6 production was detected in the DC cultures upon 24 h of CT treatment, and there was more than 30-fold increase of IL-6 production during 72 h treatment (Fig. 5A). However, we failed to detect any measurable TGF-β by ELISA in the same culture supernatant (data not shown). In an *in vivo* setting, significantly increased IL-6 production was also observed in the lungs when bronchoalveolar lavage samples from CT-treated B6 mice were examined (Fig. 5B). We also checked the levels of TGF-β in the lungs, and intriguingly, constitutively high levels of TGF-β (more than 500 pg/ml in the lung homogenates) were detected by ELISA in mice during the experimental periods (data not shown). These results strongly suggest that induction of IL-6 mediates the ability of CT inducing Th17-dominated responses. To determine whether endogenous IL-6 production is necessary for the Th17-driving ability of CT, IL-6 knockout (IL-6KO) mice were used for the experiment. Compared to CT-treated wild type mice, IL-6KO mice are almost completely resistant to the Th-17-driving effect of CT (Fig. 5C, D), indicating that IL-6 induction is required for the effect.

The previous results suggest that CT B subunit attribute to the Th17-driving effect of CT (Fig. 2). To define the role of ganglioside GM1 binding by CTB for the IL-6 induction and to exclude the possible effect of LPS contamination, CT was pre-incubated with 5-fold molar excess of GM1 and then added to DC cultures. Interestingly, saturation of GM1 binding site significantly decreased the levels of IL-6 in the supernatant throughout the culture period except for 24 h which was statistically not significant (Fig. 5E). These results indicate that GM1 receptor binding of CTB is important for the induction of IL-6 by CT. To further exclude the possible influence of LPS contamination, CT was heat-treated (100°C for 30 min) before administration into the mice. Heat-inactivation of CT almost completely abolished the induction of Th17 cells (Fig. 5F), suggesting that the observed Th17-driving activity of CT was not due to endotoxin contamination and the native conformation of CT is necessary for the activity.

## Discussion

In this study, we investigated the effect of CT on the differentiation of IL-17-producing CD4 T cells in various *in vivo* settings. CT is a powerful mucosal and/or parenteral adjuvant which enhances cellular immune responses as well as mucosal IgA and serum IgG responses to the coadministered antigens. Our results clearly demonstrate that CT, through IL-6-dependent pathway, facilitates the generation of Th17-type CD4 T cells. Th17-type T cells, which produce IL-17, IL-17F, and IL-22, are thought to be important for inflammatory responses and the control of bacterial and fungal infections at mucosal surfaces [26], [27]. Previously, the requirement of IL-17 and IL-17-producing T cells in host defense against extracellular pathogens have been well demonstrated [28]–[30]. In addition, recent studies have shown that IL-17A responses play an important role in naturally-acquired immunity to pneumococcus in humans [31], and Th17-dependent protection against pneumococcal challenge in mouse model [32]. Thus, this novel ability of CT to induce Th17-skewed responses may certainly contribute to the immunological adjuvanticity of the toxin and facilitate the development of efficacious vaccines against respiratory bacterial infections.

IL-6 and TGF-β are essential cytokines for Th17 development *in vitro* [22]–[24] and the orphan nuclear receptor RORγt, which is induced by IL-6 and TGF-β, is required for the expression of IL-17 [33]. Although Th17 cells are shown to be involved in a variety of autoimmune disease models in mice, their major role might be a defense mechanism against extracellular bacteria and fungi [34]–[36]. However, the conditions for the induction of Th17 cells by *in vivo* immunization have not been investigated yet. Thus, our observation provides the evidence that antigen-specific Th17 type responses could be actively induced with use of CT as mucosal adjuvant. As IL-17-secreting T cells were shown to be a key component of protective immunity to lung infections by bacterial pathogens [37], [38], this Th17-inducing activity of CT adjuvant might be critical for certain bacterial vaccines. In addition, we have shown that Th17-driving adjuvanticity of CT resides in the B subunit. Although CT has the most potent adjuvant activity, the use of this toxin in vaccination may pose risks due to its innate toxicity. Thus, successful use of CT as vaccine adjuvant in humans requires separation of toxicity from adjuvanticity. In this regard, our results have significance for the use of CTB as vaccine adjuvant in humans since non-toxic CT B subunit is responsible for the CT's ability to induce Th17-dominated responses. Future studies will further determine the effectiveness of antigen-specific Th17-type responses induced by CT adjuvant for the protective immunity against various pathogenic infections.

Recent studies have shown that oral immunization of CT elicits Th2-biased responses, which in turn support the development of high titer of IgG1, IgE, and mucosal IgA [3], [5]. Other studies also suggest that both Th1- and Th2-type responses are involved in the induction of mucosal IgA responses when CT is employed as mucosal adjuvant [8], [39], [40]. However, these studies have not examined the presence of IL-17-producing T cells as they preceded the identification of Th17 subset. Now, we demonstrate that mucosal immunization with CT induces Th17-dominating responses to bystander antigens as well as CTB itself. The mechanism(s) of CT to promote the induction of specific Th17 subsets is likely to involve direct influence on APCs. We found that CT treatment of dendritic cells induces strong IL-6 production, which was dependent on GM1 binding of CT B subunit, and the *in vivo* Th17-driving ability of CT requires endogenous IL-6 production (Fig. 5). These results are consistent with the reports that CT induces IL-1 and IL-6 secretion by epithelial cells and APC [41], [42]. Together, these data suggest that CT can trigger the production of proinflammatory cytokines such as IL-6 by signaling through the ganglioside GM1 receptor and subsequently induce the skewed differentiation of Th17 cells. However, we have not completely excluded the possibility that CTA translocated into the cell after GM1 binding of CTB might be also involved in the IL-6 production.

*In vitro* treatment of BM-derived DC with CT was also shown to induce full maturation as well as nuclear localization of NF-κB in DC in a GM1-dependent fashion [43], and up-regulated production of cytokines such as IL-1β [44]. It has been reported that TGF-β1 is constitutively expressed in the lungs and airway epithelial cells activate latent TGF-β1 through binding of the integrin αvβ6 which is up-regulated during pulmonary inflammation [45]. Consistent with this observation, constitutively high amounts of TGF-β1 were detected by ELISA in the lungs of CT-treated mice during the course of our experiments (data not shown). Thus, it is likely that CT activates DCs and lung epithelial cells through GM1-ganglioside binding to produce and activate Th17-driving cytokines such as IL-6 and TGF-β, respectively, which result in the skewed differentiation of Th17 cells during antigenic stimulation.

In summary, we demonstrate that nasal immunization of CT promotes the differentiation of Th17 cells directed to bystander antigens, revealing a novel adjuvanticity of CT. Our data presented here help understand the ability of CT to regulate T helper demarcation and suggest the potential implication of CT adjuvant for inducing desired types of immunity.

## Materials and Methods

### Mice and Ethics statement

Female C57BL/6 (B6) mice and BALB/c mice were purchased from Charles River Laboratories (Yokohama, Japan). OT-II TCR transgenic mice and IL-6KO mice (B6 background) were from the Jackson Laboratory (Bar Harbor, ME). Mice were housed under specific pathogen-free conditions and were used between 5 and 8 weeks of age. All animals were handled in strict accordance with good animal practice as defined by the relevant national and/or local animal welfare bodies, and all animal work was approved by Ewha Womans University's institutional animal care and use committee.

### Reagents

CT and CTB were purchased from List Biological Laboratories (Campbell, CA). For the recombinant CTA1, DNA encoding cholera toxin A1 subunit (amino acid 1-194) was amplified by PCR with the *Vibrio cholerae* (N16961 strain) DNA as a template, and cloned into Xho I and BamH I sites of the pET15b-Tat-GFP-Tat plasmid, resulting in pET15b-TCTA1T. The recombinant CTA1 was expressed and purified using Talon metal affinity column as recommended by the manufacturer (Clontech, Palo Alto, CA). Peptides were synthesized from Peptron Inc. (Daejon, Korea). CpG ODN (TCCATGACGTTCCTGACGTT) with phosphorothioate backbones was obtained from Bioneer Corp. (Daejon, Korea). PMA and ionomycin were purchased from Sigma-Aldrich (St. Louis, MO). Antibodies for flow cytometric analysis were from BioLegend (San Diego, CA) and TCR-specific anti-Vα2 (B20.1) and Vβ5 (MR9-4) were from BD Bioscience (San Diego, CA).

### Preparation of recombinant adenovirus and RSV

Recombinant defective adenovirus expressing OVA (rAd/OVA) was kindly provided by Y. C. Sung (Department of Molecular and Life science, POSTECH). The rAd/OVA stocks were propagated in 293 cells and titrated by TCID_50_. RSV A2 strain was propagated in HEp-2 cells (ATCC, Manassas, VA) in DMEM medium supplemented with 3% heat-inactivated FCS, and titrated for infectivity by plaque assay.

### Immunization

For peptide immunization, 2×10^6^ OT-II splenocytes were i.v. injected into B6 mice, and then recipient mice were i.n. immunized with 40 µg of OVA peptide plus 30 µg of CpG ODN, 0.2 µg of CT, or 2 µg of CT or CTB in PBS. For virus infection, mice were i.n. inoculated with 5×10^6^ PFU (for B6) or 1×10^6^ PFU (for BALB/c) of RSV or 5×10^7^ PFU of rAd/OVA.

### Surface staining, intracellular staining, and flow cytometric analysis

At appropriate time points, experimental mice were sacrificed, and lung tissues, spleens, and mediastinal lymph nodes were isolated. The lungs were perfused with 5 ml of PBS containing 10 U/ml heparin (Sigma-Aldrich) through the right ventricle. The tissues were then processed through a steel screen to obtain single cell suspension and particulate matter was removed by passing through 70 µm Falcon cell strainers (BD Labware). Freshly explanted cells were purified by density gradient centrifugation and resuspended in FACS buffer (1 % FBS, 0.03 % sodium azide in PBS) at a concentration of 1×10^7^ cells/ml. A total of 100 µl of these cells (1×10^6^ cells) was stained for CD8 (clone 53-6.7), CD4 (GK1.5), TCR Vα2 (B20.1), Vβ5 (MR9-4) or Gr-1(RB6-8C5) and samples were acquired on FACSCalibur™ (BD Biosciences). PE-conjugated OVA-specific MHC I tetramer, OVA-Tet, was produced as described elsewhere [46], and the optimal concentration was determined by titration. Cells were stained for 40 min at 4°C using fluorochrome-conjugate Abs and OVA-Tet, washed, and fixed in PBS containing 2% formaldehyde before analysis by flow cytometry. For intracellular staining, cells were stimulated either with 10 µM peptide or PMA(50 ng/ml)/ionomycin(500 ng/ml) for 5 h in the presence of Brefeldin A (10 µg/ml). The cells were first stained for surface markers, washed, fixed and permeabilized with FACS buffer containing 0.5% Saponin (Sigma-Aldrich). Then, the cells were stained with anti-IFN-γ (XMG1.2), IL-17A (TC11-18H10.1), IL-4 (11B11) or IL-10 (JES5-16E3). Gates were set on lymphocytes by forward and side scatter profiles, and the data were analyzed using CellQuest™ Pro (BD Biosciences) and WinMDI version 2.9 software (The Scripps Research Institute, La Jolla, CA).

### Cytokine assays

Dendritic cells (DCs) were generated from bone marrow of B6 or BALB/c mice by culturing in complete RPMI medium containing 10% FBS supplemented with 10 ng/ml recombinant GM-CSF and IL-4 (R&D, Minneapolis, MN). After 7 days of culture, non-adherent cells were harvested by gentle pipetting, and DCs were enriched by density gradient centrifugation over Percoll medium. After purification, DCs were further incubated with PBS or CT (100 ng/ml) for 3 days and the culture supernatants were harvested at the indicated time points. For blocking experiment, the cholera toxin was pre-incubated with 5-fold molar excess of GM1 ganglioside (10 ng/ml; Calbiochem, La Jolla, CA) for 30 min at 25°C before being added to the culture. The levels of specific cytokines were determined by ELISA kits for IL-6 and TGF-β (eBioscience) according to the manufacturer's protocol. Recombinant cytokine proteins were used as standards for calculating cytokine concentrations in the culture supernatants. The assays were carried out in triplicate wells.

### Statistical analysis

Two-tailed Student's t test was used for comparison of means, and values of P<0.05 were considered statistically significant.

## References

[1] Williams NA, Hirst TR, Nashar TO (1999). Immune modulation by the cholera-like enterotoxins: from adjuvant to therapeutic.. Immunol Today.

[2] Yamamoto S, Kiyono H, Yamamoto M, Imaoka K, Fujihashi K (1997). A nontoxic mutant of cholera toxin elicits Th2-type responses for enhanced mucosal immunity.. Proc Natl Acad Sci U S A.

[3] Xu-Amano J, Kiyono H, Jackson RJ, Staats HF, Fujihashi K (1993). Helper T cell subsets for immunoglobulin A responses: oral immunization with tetanus toxoid and cholera toxin as adjuvant selectively induces Th2 cells in mucosa associated tissues.. J Exp Med.

[4] Simecka JW, Jackson RJ, Kiyono H, McGhee JR (2000). Mucosally induced immunoglobulin E-associated inflammation in the respiratory tract.. Infect Immun.

[5] Marinaro M, Staats HF, Hiroi T, Jackson RJ, Coste M (1995). Mucosal adjuvant effect of cholera toxin in mice results from induction of T helper 2 (Th2) cells and IL-4.. J Immunol.

[6] Ryan EJ, McNeela E, Pizza M, Rappuoli R, O'Neill L (2000). Modulation of innate and acquired immune responses by Escherichia coli heat-labile toxin: distinct pro- and anti-inflammatory effects of the nontoxic AB complex and the enzyme activity.. J Immunol.

[7] Braun MC, He J, Wu CY, Kelsall BL (1999). Cholera toxin suppresses interleukin (IL)-12 production and IL-12 receptor beta1 and beta2 chain expression.. J Exp Med.

[8] Yanagita M, Hiroi T, Kitagaki N, Hamada S, Ito HO (1999). Nasopharyngeal-associated lymphoreticular tissue (NALT) immunity: fimbriae-specific Th1 and Th2 cell-regulated IgA responses for the inhibition of bacterial attachment to epithelial cells and subsequent inflammatory cytokine production.. J Immunol.

[9] Schaffeler MP, Brokenshire JS, Snider DP (1997). Detection of precursor Th cells in mesenteric lymph nodes after oral immunization with protein antigen and cholera toxin.. Int Immunol.

[10] Lavelle EC, McNeela E, Armstrong ME, Leavy O, Higgins SC (2003). Cholera toxin promotes the induction of regulatory T cells specific for bystander antigens by modulating dendritic cell activation.. J Immunol.

[11] Krieg AM (2002). CpG motifs in bacterial DNA and their immune effects.. Annu Rev Immunol.

[12] Cong Y, Bowdon HR, Elson CO (1996). Identification of an immunodominant T cell epitope on cholera toxin.. Eur J Immunol.

[13] Liu SJ, Tsai JP, Shen CR, Sher YP, Hsieh CL (2007). Induction of a distinct CD8 Tnc17 subset by transforming growth factor-beta and interleukin-6.. J Leukoc Biol.

[14] Intlekofer AM, Banerjee A, Takemoto N, Gordon SM, Dejong CS (2008). Anomalous type 17 response to viral infection by CD8+ T cells lacking T-bet and eomesodermin.. Science.

[15] Lukens MV, Claassen EA, de Graaff PM, van Dijk ME, Hoogerhout P (2006). Characterization of the CD8+ T cell responses directed against respiratory syncytial virus during primary and secondary infection in C57BL/6 mice.. Virology.

[16] Sacks D, Noben-Trauth N (2002). The immunology of susceptibility and resistance to Leishmania major in mice.. Nat Rev Immunol.

[17] Guinazu N, Pellegrini A, Giordanengo L, Aoki MP, Rivarola HW (2004). Immune response to a major Trypanosoma cruzi antigen, cruzipain, is differentially modulated in C57BL/6 and BALB/c mice.. Microbes Infect.

[18] Ferretti S, Bonneau O, Dubois GR, Jones CE, Trifilieff A (2003). IL-17, produced by lymphocytes and neutrophils, is necessary for lipopolysaccharide-induced airway neutrophilia: IL-15 as a possible trigger.. J Immunol.

[19] Schwarzenberger P, La Russa V, Miller A, Ye P, Huang W (1998). IL-17 stimulates granulopoiesis in mice: use of an alternate, novel gene therapy-derived method for in vivo evaluation of cytokines.. J Immunol.

[20] Witowski J, Pawlaczyk K, Breborowicz A, Scheuren A, Kuzlan-Pawlaczyk M (2000). IL-17 stimulates intraperitoneal neutrophil infiltration through the release of GRO alpha chemokine from mesothelial cells.. J Immunol.

[21] Fleming TJ, Fleming ML, Malek TR (1993). Selective expression of Ly-6G on myeloid lineage cells in mouse bone marrow. RB6-8C5 mAb to granulocyte-differentiation antigen (Gr-1) detects members of the Ly-6 family.. J Immunol.

[22] Mangan PR, Harrington LE, O'Quinn DB, Helms WS, Bullard DC (2006). Transforming growth factor-beta induces development of the T(H)17 lineage.. Nature.

[23] Bettelli E, Carrier Y, Gao W, Korn T, Strom TB (2006). Reciprocal developmental pathways for the generation of pathogenic effector TH17 and regulatory T cells.. Nature.

[24] Veldhoen M, Hocking RJ, Atkins CJ, Locksley RM, Stockinger B (2006). TGFbeta in the context of an inflammatory cytokine milieu supports de novo differentiation of IL-17-producing T cells.. Immunity.

[25] Gagliardi MC, Sallusto F, Marinaro M, Langenkamp A, Lanzavecchia A (2000). Cholera toxin induces maturation of human dendritic cells and licences them for Th2 priming.. Eur J Immunol.

[26] Liang SC, Tan XY, Luxenberg DP, Karim R, Dunussi-Joannopoulos K (2006). Interleukin (IL)-22 and IL-17 are coexpressed by Th17 cells and cooperatively enhance expression of antimicrobial peptides.. J Exp Med.

[27] Harrington LE, Mangan PR, Weaver CT (2006). Expanding the effector CD4 T-cell repertoire: the Th17 lineage.. Curr Opin Immunol.

[28] Huang W, Na L, Fidel PL, Schwarzenberger P (2004). Requirement of interleukin-17A for systemic anti-Candida albicans host defense in mice.. J Infect Dis.

[29] Higgins SC, Jarnicki AG, Lavelle EC, Mills KH (2006). TLR4 mediates vaccine-induced protective cellular immunity to Bordetella pertussis: role of IL-17-producing T cells.. J Immunol.

[30] Khader SA, Bell GK, Pearl JE, Fountain JJ, Rangel-Moreno J (2007). IL-23 and IL-17 in the establishment of protective pulmonary CD4+ T cell responses after vaccination and during Mycobacterium tuberculosis challenge.. Nat Immunol.

[31] Lu YJ, Gross J, Bogaert D, Finn A, Bagrade L (2008). Interleukin-17A mediates acquired immunity to pneumococcal colonization.. PLoS Pathog.

[32] Bogaert D, Weinberger D, Thompson C, Lipsitch M, Malley R (2009). Impaired innate and adaptive immunity to Streptococcus pneumoniae and its effect on colonization in an infant mouse model.. Infect Immun.

[33] Ivanov, McKenzie BS, Zhou L, Tadokoro CE, Lepelley A (2006). The orphan nuclear receptor RORgammat directs the differentiation program of proinflammatory IL-17+ T helper cells.. Cell.

[34] Huang W, Na L, Fidel PL, Schwarzenberger P (2004). Requirement of interleukin-17A for systemic anti-Candida albicans host defense in mice.. J Infect Dis.

[35] Happel KI, Dubin PJ, Zheng M, Ghilardi N, Lockhart C (2005). Divergent roles of IL-23 and IL-12 in host defense against Klebsiella pneumoniae.. J Exp Med.

[36] Chung DR, Kasper DL, Panzo RJ, Chitnis T, Grusby MJ (2003). CD4+ T cells mediate abscess formation in intra-abdominal sepsis by an IL-17-dependent mechanism.. J Immunol.

[37] Priebe GP, Walsh RL, Cederroth TA, Kamei A, Coutinho-Sledge YS (2008). IL-17 is a critical component of vaccine-induced protection against lung infection by lipopolysaccharide-heterologous strains of Pseudomonas aeruginosa.. J Immunol.

[38] Higgins SC, Jarnicki AG, Lavelle EC, Mills KH (2006). TLR4 mediates vaccine-induced protective cellular immunity to Bordetella pertussis: role of IL-17-producing T cells.. J Immunol.

[39] Kurono Y, Yamamoto M, Fujihashi K, Kodama S, Suzuki M (1999). Nasal immunization induces Haemophilus influenzae-specific Th1 and Th2 responses with mucosal IgA and systemic IgG antibodies for protective immunity.. J Infect Dis.

[40] Imaoka K, Miller CJ, Kubota M, McChesney MB, Lohman B (1998). Nasal immunization of nonhuman primates with simian immunodeficiency virus p55gag and cholera toxin adjuvant induces Th1/Th2 help for virus-specific immune responses in reproductive tissues.. J Immunol.

[41] McGee DW, Elson CO, McGhee JR (1993). Enhancing effect of cholera toxin on interleukin-6 secretion by IEC-6 intestinal epithelial cells: mode of action and augmenting effect of inflammatory cytokines.. Infect Immun.

[42] Bromander AK, Kjerrulf M, Holmgren J, Lycke N (1993). Cholera toxin enhances alloantigen presentation by cultured intestinal epithelial cells.. Scand J Immunol.

[43] Kawamura YI, Kawashima R, Shirai Y, Kato R, Hamabata T (2003). Cholera toxin activates dendritic cells through dependence on GM1-ganglioside which is mediated by NF-kappaB translocation.. Eur J Immunol.

[44] Eriksson K, Fredriksson M, Nordstrom I, Holmgren J (2003). Cholera toxin and its B subunit promote dendritic cell vaccination with different influences on Th1 and Th2 development.. Infect Immun.

[45] Munger JS, Huang X, Kawakatsu H, Griffiths MJ, Dalton SL (1999). The integrin alpha v beta 6 binds and activates latent TGF beta 1: a mechanism for regulating pulmonary inflammation and fibrosis.. Cell.

[46] Chang J, Cho JH, Lee SW, Choi SY, Ha SJ (2004). IL-12 priming during in vitro antigenic stimulation changes properties of CD8 T cells and increases generation of effector and memory cells.. J Immunol.

